# Flexible Piezoresistive Sensors from Polydimethylsiloxane Films with Ridge-like Surface Structures

**DOI:** 10.3390/mi14101940

**Published:** 2023-10-18

**Authors:** Ming Liu, Xianchao Liu, Fuqian Yang

**Affiliations:** 1 Fujian Provincial Key Laboratory of Terahertz Functional Devices and Intelligent Sensing, School of Mechanical Engineering and Automation, Fuzhou University, Fuzhou 350108, China; 210227010@fzu.edu.cn; 2 The Engineering Research Center for CAD/CAM of Fujian Universities, Putian University, Putian 351100, China; 3 State Key Laboratory of Digital Manufacturing Equipment and Technology, School of Mechanical Science and Engineering, Huazhong University of Science and Technology, Wuhan 430074, China; 4 Materials Program, Department of Chemical and Materials Engineering, University of Kentucky, Lexington, KY 40506, USA

**Keywords:** flexible sensor, PDMS, surface structure, bio-applications

## Abstract

Developing flexible sensors and actuators is of paramount importance for wearable devices and systems. In this research, we developed a simple and facile technique to construct flexible piezoresistive sensors from polydimethylsiloxane films with ridge-like surface structures and laser-induced porous graphene. Using a replication strategy, we prepared the ridge-like surface structures from sandpapers. The piezoresistive sensors exhibit excellent sensitivity with a response time of less than 50 ms and long-term cyclic stability under mechanical loading. The smallest weight they can sense is ~96 mg. We demonstrated applications of the piezoresistive sensors in the sensing of bio-related activities, including muscle contraction, finger flexion, wrist flexion, elbow bending, knee bending, swallowing, respiration, sounds, and pulses.

## 1. Introduction

The increasing attention to health monitoring and wearable devices, which have considerable market prospects in human–computer interactions [1,2], has stimulated extensive research to develop flexible and wearable devices for medical monitoring [3,4,5] and motion detection [6], due to their portable and timely response capabilities. Among various mechanisms for flexible and wearable devices, pressure sensing has attracted great interest. Generally, the mechanisms of pressure sensing can be categorized into four groups: piezoresistive response [7], capacitive response [8,9,10], piezoelectric response [11,12,13], and triboelectric response [14,15]. Among the mechanisms of pressure sensing, piezoresistive sensing has the advantages of simple preparation, easy signal readout, and high linearity [16,17].

Various materials, including MXene [18,19,20], metal nanowires [21], carbon nanotubes [22,23], graphene [24,25,26], poly(3,4-ethylenedioxythiophene) polystyrene sulfonate (PEDOT:PSS) [27], reduced graphene oxide (rGO) [28], carbon black (CB) [29], carbon ink [16], and metal nanoparticles [30,31], have been explored for applications in flexible structures and pressure sensors. Among these materials, graphene has exhibited unique characteristics with its large specific surface area and high electrical conductivity. The applications of graphene in flexible electronic devices have been cultivated by the laser-induced formation of porous graphene (LIG) from commercially available polymers such as polyimide (PI) [32]. LIG, which is produced at low cost and without production of toxic gas compared to conventional methods to produce graphene, exhibits a three-dimensional porous structure, and has been widely recognized as an effective material for applications in flexible piezoresistive sensors [26,33,34,35]. Engineering geometrical characteristics, such as micro-cones [36,37,38], micro-domes [39,40,41,42], microgrooves [43], micro-columnar arrays [9,44,45], natural paper [16], and pleated structures [12,46] between the sensitive layer and the electrodes, allows for the increase in initial resistance especially on surfaces of polymers such as polydimethylsiloxane (PDMS) [47,48], PI [49], and Ecoflex [50,51], and can improve the sensitivity of flexible piezoresistive sensors.

Tian et al. [52] constructed a sensor with a layer of microspheres sandwiched between two LIG/polyurethane membranes, and found a sensitivity of 149 kPa^−1^ and a minimum detection weight of 20 mg in the low-pressure range. Converting natural wood into rGO-modified flexible wood (FW/rGO), Guan et al. [53] prepared wood-based flexible piezoresistive pressure sensors with a sensitivity of 1.85 kPa^−1^ at a pressure of 60 kPa. Wang et al. [54] developed a flexible haptic sensor with a pressure sensitivity of 0.58 kPa^−1^ in a pressure range of 0.3~0.4 kPa, using biomimetic micropatterned PDMS replicated from a lotus leaf. Cheng et al. [55] constructed a sensor from a microcone structure with an etching method, although their method is complex and expensive, and is not eco-friendly. Spraying AgNWs on the surface of sandpaper-molded PDMS, Jia et al. [56] prepared a sensor with a sensitivity of 0.1194 kPa^−1^ and a response time of 50 ms in the low-pressure range. Using the self-healing PDMS elastomer-functionalized ureido and imido and sandpaper pre-sprayed with uracil-grafted carbon nanotubes/polyurea mixture, Yang et al. [57] prepared piezoresistive sensors with a detection limit of 50 Pa, a fast response time of 40 ms, and a sensitivity of 0.087 kPa^−1^ in the low-pressure range.

Realizing the potential role of surface structures in enhancing the sensitivity and performance of flexible sensors and the possibility of using an inverted mold method to engineer surface structures, we constructed high-performance flexible piezoresistive sensors from PDMS films with ridge-like surface structures. The ridge-like surface structure on a PDMS film can allow for the formation of stable and homogeneous gaps for air between the sensing layer formed on the surface of the PDMS film and the electrode. Graphene was used to form the sensing layer, and LIG was used as the electrodes. The sensitivity of the pressure sensors is 0.1667 kPa^−1^ in the low-pressure range of 0–2 kPa, and the response times to loading and unloading were 47 ms and 8 ms, respectively. The minimum sensing weight was 96 mg, and the sensors maintained excellent performance after 5000 cycles of endurance testing. The sensors have a variety of potential applications, including the detection of human physiological signals (pulses, respiration) and the monitoring of joint flexion and extension signals.

## 2. Construction of Flexible Sensors

**Figure 1:**
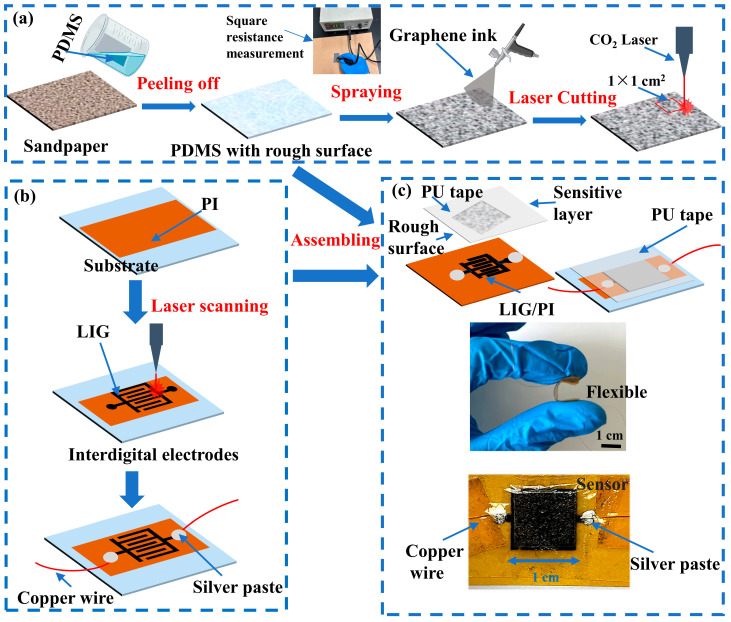
Flow chart for the preparation of a piezoresistive sensor from a PDMS film with a ridge-like surface structure: (**a**) PDMS film with a sensing layer, (**b**) LIG interdigital electrodes, and (**c**) assembling of a piezoresistive sensor.

A replication strategy was used to form a ridge-like surface structure on a PDMS film, as shown in Figure 1a. Briefly, a homogeneous PDMS mixture, which consists of a prepolymer and a cross-linking curing agent in a weight ratio of 10:1 from a commercially elastomeric PDMS kit, Sylgard 184 (Dow Corning, Midland, TX, USA), was prepared and degassed in a vacuum oven at room temperature for 10 min. The degassed mixture was cast on the rough surface of a sandpaper to form a film. The free surface of the film was smoothed via a wet-film coater to achieve ~100 μm in thickness. Placing the structure with the film and the sandpaper in an oven at 70 °C for 1 h allowed for the formation of a PDMS film. After removing the structure from the oven, the PDMS film was slowly and gently peeled from the sandpaper, and a ridge-like structure on one of the surfaces of the PDMS film was achieved.

A graphene ink was made from an oily graphene conductive slurry (Nanjing XFNANO Materials Tech Company Ltd., Nanjing, China) and ethanol (Shanghai, Aladdin Biochemical Technology Co., Ltd., Shanghai, China) was prepared. To control the uniformity and thickness of the sprayed graphene ink, the resistance of the square conductive (or sensitive) layer was measured using a coated square resistance tester. The graphene ink was sprayed onto the PDMS surface with a ridge-like structure, which was dried in an oven at 60 °C for 50 min. Multiple spraying and drying procedures were performed until the resistance reached a value in a range of 40 to 60 Ω. We used a CO_2_ laser cutting machine (FALASER Corporation) to cut the PDMS film with the layer of graphene into multiple pieces of 1 × 1 cm^2^. Cleaning up of the prepared 1 × 1 cm^2^ PDMS films was conducted to remove the silica (SiO_2_) particles generated at the edges of the PDMS films by laser cutting. Note that the SiO_2_ particles have little effect on the sensitivity of the prepared sensors, which is mainly dependent on the variations in the contact between the sensitive layer and the electrode under external loading. Five different sandpapers of 60, 150, 220, 400, and 600 grits were used to form different ridge-like surface structures to examine the effects of the ridge size. The average sizes of the SiC particles are 250, 93, 68, 23.6, and 16 μm for the sandpapers of 60, 150, 220, 400, and 600 grits, respectively.

Figure 1b shows schematically the process used to fabricate LIG. Briefly, a glass plate was cleaned first with alcohol, and a PI film of 20 × 40 mm^2^ was cut from a KAPTON PI tape (Longhui Industry Co., Ltd., Shenzhen, China) of 50 μm in thickness. The PI film was attached to the surface of the glass plate. The laser beam from a CO_2_ laser engraver was used to produce LIG interdigital electrodes on the PI film under a laser power of 6.8 W at a scanning speed of 200 mm/s to form a porous structure instead of a fibrous structure [58]. Note that the quality of the LIG is dependent on the laser power and the scanning speed. A PI film with porous LIG and a PDMS with a graphene layer (the sensing layer) were held together using medical PU (polyurethane) tape. The adhesion between the PI film and the PDMS film only played a small role in the initial contact between the porous LIG and the graphene layer without the action of compression. The contact area between the porous LIG and the graphene layer increased with the increase in compression/pressure, and the contact was reversible during unloading. Silver paste was used to connect the LIG interdigital electrodes to copper wires.

To minimize possible inconsistencies during assembling, we placed the PDMS film with the sensing layer in the center of a PU tape, which was then aligned with the LIG interdigital electrodes to form a sandwich structure. Finally, the structure was compressed together with fingers along the edges of the PDMS film rather than on the PDMS film. Note that the structure should not be compressed directly by a finger. The graphene layer was immobile after releasing the compression around the edges of the assembled structure.

## 3. Results and Discussion

**Figure 2:**
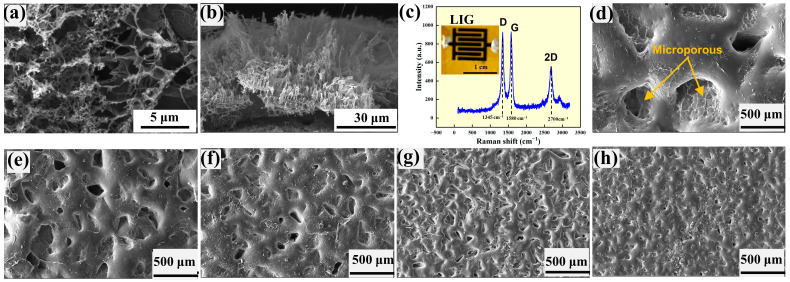
SEM images and Raman spectrum of LIG interdigital electrodes: (**a**) top surface, (**b**) cross section, and (**c**) Raman spectrum (The D peak is the disordered vibrational peak of graphene, the G peak represents the E_2g_ mode with double degenerate centers associated with the symmetry and order of graphene, and the 2D peak is the second-order Raman peak of the double phonon resonance); SEM images of the sensing layers formed on ridge-like surface structures prepared with sandpapers of different average particle sizes: (**d**) 250 μm, (**e**) 93 μm, (**f**) 68 μm, (**g**) 23.6 μm, and (**h**) 16 μm.

Both the LIG interdigital electrodes and the sensing layer were analyzed on a scanning electron microscope (SEM) (Quanta 250, FEI Inc., Hillsboro, OR, USA). Figure 2a,b present SEM images of the top surface and cross-section of an LIG. It is evident that the LIG exhibits a 3D porous structure with an average pore size of ~5 μm for the top surface. Note that the porous structure of the top surface is significantly different from the cross-section, which is present in the porous flocculent structure of ~50 μm in thickness. Such a large difference in the porous structures suggests an anisotropic effect of the laser irradiation in forming porous structures in PI. Figure 2c shows the Raman spectrum of the LIG taken on an Invia Reflex Raman microscope (Renishaw, New Mills, UK) under a wavelength of 532 nm. There are three peaks centered at 1345, 1580, and 2700 cm^−1^, corresponding to the D peak, G peak, and 2D peak of graphene [58], respectively. This result confirms that the LIG is graphene. The D peak is the disordered vibrational peak of graphene due to the defects in graphene. The G peak represents the *E*_2g_ mode of the double degenerate center associated with the symmetry and ordering of graphene. The 2D peak is the second-order Raman peak of double phonon resonance, which reflects the stacking mode of carbon atoms. The intensity ratios of *I*_D_/*I*_G_ and *I*_2D_/*I*_G_ (*I* is calculated from the integration over the area of the corresponding peak; the subscripts correspond to the specific peaks of D, G, and 2D) represent the defect density and the stacking mode of carbon atoms in graphene, respectively [32]. The numerical values of *I*_D_/*I*_G_ and *I*_2D_/*I*_G_ of the prepared LIG are ~0.96 and ~0.61, respectively, which are in accord with the results reported by Liu et al. [58].

The topologies of the graphene-covered ridge-like surface structures prepared from the sandpapers of 250, 93, 68, 23.6, and 16 μm in average particle sizes are shown in Figure 2d–h, respectively. The “pore” size decreases with the decrease in the average particle size (the increase in the grit number), as expected. Note that the surface of the non-porous region is relatively smooth. This allows for good contact between the sensing layer and the LIG interdigital electrodes for detecting local contact induced by an external force.

The sensitivity of a piezoresistive sensor is calculated as *S* = (Δ*R*/*R*_0_)/Δ*P*, where *R*_0_ is the resistance of the piezoresistive sensor without external loading, Δ*R* is the change in the resistance caused by the change in applied pressure Δ*P*, and *P* is the pressure applied to the sensor over a nominal contact area of 1 cm^2^. Δ*R* (= *R*_0_ − *R*) is generally positive due to the decrease in *R* with the increase in applied pressure. However, Δ*R* can be negative in some scenarios with unstable air gap structures.

**Figure 3:**
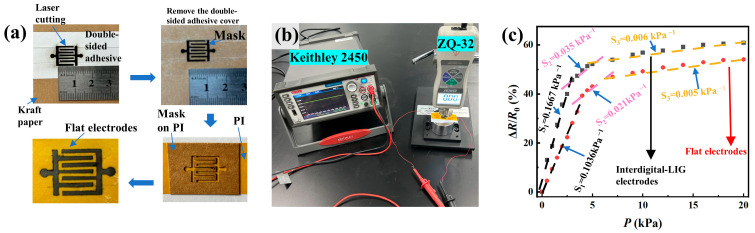
(**a**) Preparation process of planar electrodes by spraying graphene ink on PI with mask. (**b**) Optical image of the test platform for measuring the sensitivity of the prepared piezoresistive sensors under mechanical compression (digital source meter: Keithley 2450; uniaxial compression tester: ZQ-32). (**c**) Comparison of the sensitivities of the piezoresistive sensors with LIG electrodes and planar electrodes. The same sensitive layer was prepared using sandpaper with an average size of 93 μm.

To demonstrate the advantages of the LIG interdigital electrodes over planar (or flat) electrodes, planar electrodes were prepared. The preparation process is shown in Figure 3a. Firstly, a laser was used to cut double-sided adhesive tape on kraft paper, which was manually lifted off. The mask was placed on PI adhesive tape, whose surface was sprayed uniformly with graphene ink. The structure was dried in an oven at 60 °C for 50 min. The spraying process was repeated several times to achieve a linear resistance of less than 100 Ω/cm for flexible flat electrodes. Then, the mask was peeled off from the PI tape to obtain planar electrodes. Figure 3b shows the setup used to measure the resistance change in the prepared piezoresistive sensors under mechanical compression. Figure 3c shows the comparison of the sensitivities of the sensors with LIG interdigital electrodes and with the planar electrodes. In this study, the sensing layer was prepared using sandpaper with an average size of 93 μm. It is evident that the sensitivity of the piezoresistive sensor with the LIG interdigital electrodes is higher than that with the planar electrodes. Such a difference can be attributed to the porous structure of the LIG interdigital electrodes, which contributes to the increase in the sensitivity of the piezoresistive sensor. Also, the LIG interdigital electrodes were prepared via one-step lasering from carbon-based materials without any masks, which is low-cost and environmentally friendly. The electrodes have good chemical stability and high electrical conductivity [20].

**Figure 4:**
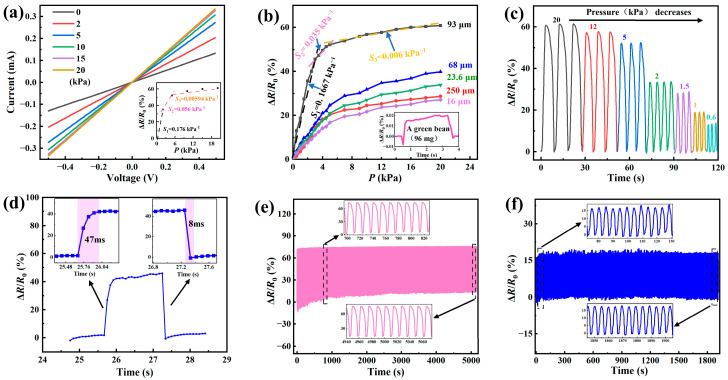
Performance of the piezoresistive sensors: (**a**) *I*–*V* curves of a piezoresistive sensor made from PMDS film with ridge-like surface structure prepared with sandpaper of 93 μm in average particle size under different pressures (the inset shows variations in Δ*R*/*R*_0_ with the applied pressure); (**b**) variations in Δ*R*/*R*_0_ with the applied pressure for the piezoresistive sensors made from PDMS films with different ridge-like surface structures (the inset shows the response of a piezoresistive sensor after placing a green bean of ~96 mg on the top of the piezoresistive sensor); (**c**) temporal evolution of Δ*R*/*R*_0_ of the piezoresistive sensor under cyclic loading and unloading for different maximum pressures; (**d**) response of the piezoresistive sensor to a single loading and unloading event by a finger; (**e**) temporal evolution of Δ*R*/*R*_0_ for the long-term stability of the piezoresistive sensor subjected to 5000 cycles of loading and unloading at a maximum pressure of 20 kPa; and (**f**) temporal evolution of Δ*R*/*R*_0_ for the long-term stability of the piezoresistive sensor subjected to 2000 cycles of loading and unloading at a maximum pressure of 1 kPa.

A mechanical–electrical system was constructed to assess the performance of the prepared piezoresistive sensor. Figure 4a shows *I*–*V* (current–voltage) curves under different pressures for the piezoresistive sensor with the ridge-like surface structure prepared with the sandpaper with an average particle size of 93 μm. The voltage is in a range of −0.5 to 0.5 V. It is interesting to note that electric current is proportional to electric voltage for voltages in the range of −0.5 to 0.5 V for the applied pressure, exhibiting Ohmic characteristics. From the *I*–*V* curves, we calculated the resistance of the piezoresistive sensor from the slope (=*V*/*I*) and analyzed variations in the ratio of Δ*R*/*R*_0_ with the applied pressure. The inset shows variations in the ratio of Δ*R*/*R*_0_ with the applied pressure *P* for the piezoresistive sensor, which can be generally divided into three ranges of small pressures, intermediate pressures, and high pressures. In the range of small pressures, there is a rapid increase in the ratio of Δ*R*/*R*_0_ with increasing the applied pressure, which is likely due to the increase in the number of multi-asperity contacts. In the range of intermediate pressures, the increase rate of the ratio of Δ*R*/*R*_0_ with respect to the applied pressure gradually decreases, likely due to the few multi-asperities available to increase the number of multi-asperity contacts for new conducting pathways; the increase in the contact area between the LIG interdigital electrodes and the sensing layer starts to play a role in the decrease in the resistance of the piezoresistive sensor. In the range of high pressures, the ratio of Δ*R*/*R*_0_ increases linearly with the applied pressure, likely due to the dominant role of the increase in the contact area between the LIG interdigital electrodes and the sensing layer without further increases in conducting pathways, which is similar to the result reported by Wang et al. [42]. Using these data, we obtained the sensitivity *S* of the piezoresistive sensor to be ~0.0176, ~0.056, and ~0.00594 kPa^−1^ for the pressure in the range of 0 to 2 kPa, 2 to 5 kPa, and 5 to 20 kPa, respectively. It is worth noting that one should use percentages in analyzing the sensitivity. The piezoresistive sensor exhibits the largest sensitivity under low pressures, suggesting the important role of the increase in conducting pathways in the sensitivity of the piezoresistive sensors made from PDMS films with ridge-like surface structures.

Figure 4b presents the variations in Δ*R*/*R*_0_ with the applied pressure for the piezoresistive sensors made from PDMS films with different ridge-like surface structures. It is evident that the variations in the ratio of Δ*R*/*R*_0_ with the applied pressure *P* can be generally divided into three regions of small pressures, intermediate pressures, and high pressures for all of the piezoresistive sensors, independent of the ridge-like surface structures. In the range of small pressures, there is a rapid increase in the ratio of Δ*R*/*R*_0_ with increasing applied pressure; in the range of intermediate pressures, the increase rate of the ratio of Δ*R*/*R*_0_ with respect to the applied pressure gradually decreases; and in the range of high pressures, the ratio of Δ*R*/*R*_0_ increases linearly with the applied pressure. Under the same applied pressure, the ratio of Δ*R*/*R*_0_ increases with the increase in the average pore/particle size (the decrease in the grit number), except the one from the PDMS films with the ridge-like surface structure made with the sandpaper of 250 μm in average particle size. The mechanism for such a difference is unclear. It may be associated with large contact areas between the asperities and the LIG interdigital electrodes. However, these results indeed indicate that the ridge-like surface structures play an important role in determining the resistance and the sensitivity of the piezoresistive sensors.

The inset in Figure 4b depicts the response behavior of a piezoresistive sensor, which was made from the PMDS film with the ridge-like surface structure prepared with the sandpaper of 93 μm in average particle size, when a green bean of ~96 mg was slowly placed on the piezoresistive sensor. There are significant changes in the ratio of Δ*R*/*R*_0_ at both the onset and the end of the action, which demonstrate an excellent sensitivity of the piezoresistive sensor to external loading. This result suggests potential applications as biosensors, including the sensing of pulse, respiration, and throat vibrations.

A test system consisting of a uniaxial tester (CMT5305) and a digital source meter (Keithley 2450) was constructed to examine the response of the piezoresistive sensors under cyclic loading and unloading. The uniaxial tester controlled by a computer was used to apply cyclic loading to the piezoresistive sensors; the digital source meter was used to record the variations in resistance of the piezoresistive sensors under cyclic loading and unloading. The digital source meter was connected to the electrodes of the piezoresistive sensor, which was fixed by a double-sided adhesive.

Figure 4c presents the temporal evolution in the ratio of Δ*R*/*R*_0_ under cyclic loading and loading of different maximum pressures for the piezoresistive sensor made from the PDMS film with the ridge-like surface structure prepared with the sandpaper of 93 μm in average particle size. Note that only three sequences of cyclic loading and unloading for each maximum pressure were applied to the piezoresistive sensor. The piezoresistive sensor exhibits excellent repeatability for each loading–unloading condition, and sensitivity to the cyclic loading and unloading.

The response behavior of the piezoresistive sensor, which was made from the PMDS films with the ridge-like surface structure prepared with the sandpaper of 93 μm in average particle size, is shown in Figure 4d for a single loading and unloading event by a finger. There is a rapid increase in the ratio of Δ*R*/*R*_0_ at the onset of the loading with a response time of ~47 ms for the pressure to reach the maximum, and a rapid decrease in the ratio of Δ*R*/*R*_0_ at the end of the unloading with a response time of ~8 ms. Such short response times suggest excellent performance of the piezoresistive sensor as well as the potential for applying it to real-time sensing of human body movements.

Figure 4e,f present the long-term stability of the piezoresistive sensor that was made from the PMDS film with the ridge-like surface structure prepared with the sandpaper of 93 μm in average particle size, under cyclic loading and unloading for the maximum pressures of 1 kPa and 20 kPa, respectively. The cycling frequency was 0.08 Hz. For both the cyclic loading and unloading, there exists a period at the beginning in which the ratio of Δ*R*/*R*_0_ is negative. Such a trend is likely due to some asperities being separated from the LIG interdigital electrodes after the unloading, leading to the loss of conducting pathways and the increase in resistance. Note that similar behavior was also reported by Xia et al. [20]. The temporal evolution of the ratio of Δ*R*/*R*_0_ becomes stable under further cyclic loading and unloading, demonstrating the long-term durability and stability of the piezoresistive sensor. Table 1 shows a comparison of the performance of different pressure sensors prepared with sandpapers as replicated structures with different sensing materials. For low pressures, the sensitivity of the piezoresistive sensors with graphene as the sensing material in this study is greater than those of the piezoresistive sensors with AgNWs [56], nanotubes [57], and MWCNTs/CNTs [59] as the sensing material, and less than those of the piezoresistive sensors with CNTs [60,61] and MXene [62] as the sensing material.

**Figure 5:**
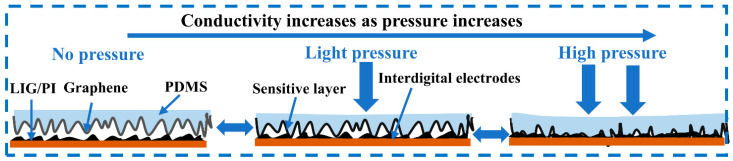
Working principle of the piezoresistive sensors.

Figure 5 illustrates the working principle of the piezoresistive sensors. The irregular ridge-like surface allows for the formation of multiple voids between the sensing layer and the LIG interdigital electrodes. The number of conducting pathways is dependent on the number and sizes of the multi-asperity contacts between the sensing layer and the LIG interdigital electrodes, which increase with the increase in pressure on the piezoresistive sensor. Increasing the number and sizes of the multi-asperity contacts reduces the resistance between the sensing layer and the LIG interdigital electrodes. At low pressures, the number and sizes of the multi-asperity contacts determine the sensitivity of the sensor; at high pressures, the sizes of the multi-asperity contacts mainly determine the sensitivity of the piezoresistive sensor, since there are few asperities available to increase the number of multi-asperity contacts, and the sizes of the multi-asperity contacts are dependent on contact deformation. The elastic characteristics of PDMS allow for the recovery of the piezoresistive sensors to the corresponding initial states after complete unloading.

Using the piezoresistive sensors that were made from the PMDS film with the ridge-like surface structure prepared with sandpaper of 93 μm in average particle size, we explored a variety of applications in the following section.

## 4. Applications of the Piezoresistive Sensors

All physiological activities, such as breathing, heartbeat, blood pressure, joint movement, and so forth, generate unique signals, which can be monitored by flexible sensors and are critical for early detection in healthcare, such as the early diagnosis, treatment, and prevention of flat feet, for Parkinson’s disease, and for vascular and cardiovascular diseases. Lu et al. [63] realized gait monitoring by integrating flexible sensors into sensor arrays to measure plantar pressure distribution. Chen et al. [64] prepared flexible pressure sensors with excellent performance, and the sensors successfully distinguished three characteristic waves of the pulses; this can be used for noninvasive blood pressure monitoring without a cuff.

**Figure 6:**
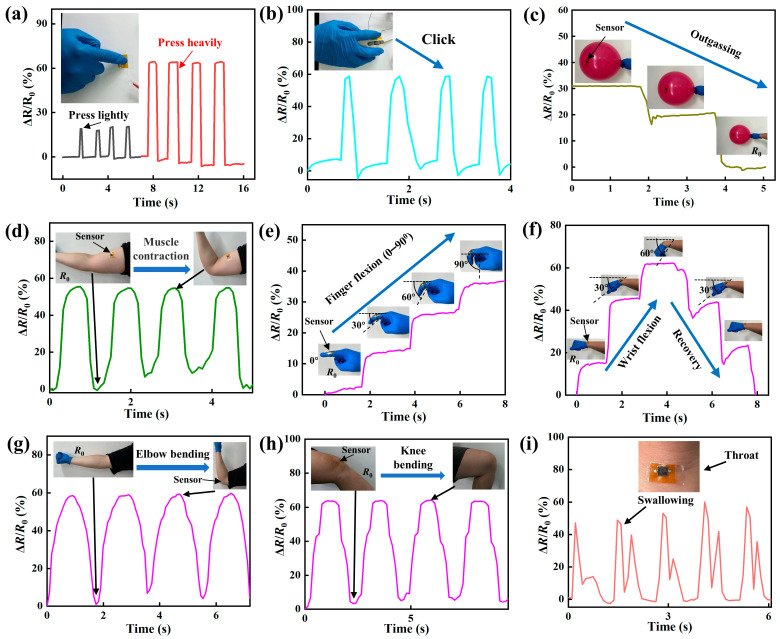
Temporal variations in the ratio of Δ*R*/*R*_0_ for the responses of the piezoresistive sensors to various motions: (**a**) finger press, (**b**) click of a mouse button, (**c**) outgassing of an air-filled balloon, (**d**) contraction of arm muscle, (**e**) finger flexion (the sensor is attached to the second joint of the index finger), (**f**) wrist flexion (the sensor is attached to the wrist), (**g**) elbow bending, (**h**) knee bending, and (**i**) swallowing of throat.

The responses of the piezoresistive sensors to a variety of mechanical loading scenarios were evaluated. Figure 6a shows temporal variations in the ratio of Δ*R*/*R*_0_ of a piezoresistive sensor to the press of a finger under different loads (light and heavy pressure). For both of the loadings, there was a rapid increase and decrease in the ratio of Δ*R*/*R*_0_ at the onset and end of the press, respectively. This result demonstrates excellent sensitivity of the piezoresistive sensor to quasi-static loading by an external object.

A piezoresistive sensor was attached to the button of a computer mouse. The temporal variations in the ratio of Δ*R*/*R*_0_, as shown in Figure 6b, represents the response of the piezoresistive sensor to the mouse clicking. There is a period with a “gradual” increase in the ratio of Δ*R*/*R*_0_, revealing the “slow” increase in the external load during the clicking. The duration in which the ratio of Δ*R*/*R*_0_ is a maximum is smaller than the quasi-static loading by an external object shown in Figure 6a. Such differences are due to the “dynamic” characteristics of mouse clicking.

To examine if the prepared piezoresistive sensors are sensitive to the deformation induced by the swelling and contraction of soft matter, a piezoresistive sensor was attached to the surface of a balloon filled with air. The responses of the piezoresistive sensor to three different states of the balloon during outgassing are illustrated by the changes in the ratio of Δ*R*/*R*_0_, with *R*_0_ as the resistance with the least air in the balloon. Increasing the balloon radius (the pressure in the balloon) increases the ratio of Δ*R*/*R*_0_, demonstrating the feasibility of using the prepared piezoresistive sensors in sensing swelling and contraction of soft matter. Such behavior can be attributed to changes in both the number of conducting pathways and the contact sizes between the LIG interdigital electrodes and the multi-asperities.

The prepared piezoresistive sensors were used to sense and monitor real-time movements of the human body at various positions, including the arm, finger, wrist, elbow, knee, and throat, as shown in Figure 6d–i, respectively. The piezoresistive sensors exhibit excellent sensitivity and stability under dynamic loading, as supported by the temporal variations in the ratio of Δ*R*/*R*_0_ at different deformation states, including tension, contraction, bending, and swallowing. It is interesting to note that there are two “pulses” per swallowing cycle of the throat (Figure 6i), which can be attributed to the up and down motions of the laryngeal node that exerts force on the piezoresistive sensor. Thus, the piezoresistive sensors can be likely used to diagnose and monitor dysphagia.

**Figure 7:**
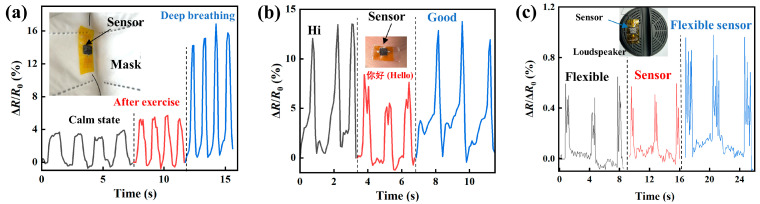
Temporal variations in the ratio of Δ*R*/*R*_0_ for the responses of the piezoresistive sensors to respiration and sounds. (**a**) Three different breathing states (calm, after 3 min of jogging, deep breathing). The inset shows that a piezoresistive sensor is attached to a mask. (**b**) Relative change in the ratio of Δ*R*/*R*_0_ under three different vocal sounds (i.e., pronunciations of “Good”, “你好 (Hello in Chinese)”, and “Hi”). The inset shows that the flexible sensor is attached to the throat of a volunteer. (**c**) The relative change in the ratio of Δ*R*/*R*_0_ under different acoustic vibrations by playing different words (i.e., “flexible”, “sensor”, and “flexible sensor”). The inset shows that the sensor is attached to a loudspeaker.

The responses of the prepared piezoresistive sensors to respiration and sounds are illustrated. A piezoresistive sensor was attached to a mask that was worn by a volunteer. Figure 7a shows the response of the piezoresistive sensor to respirations of the volunteer at three different states of calm, after exercise, and deep breathing. Significant differences in the ratio of Δ*R*/*R*_0_ are observed, including the rate and magnitude of the ratio of Δ*R*/*R*_0_.

The changes in the ratio of Δ*R*/*R*_0_ are presented in Figure 7b, corresponding to the response of a piezoresistive sensor that was attached to the throat of a volunteer to the sounds of different words. It is evident that the temporal variations in Δ*R*/*R*_0_ for different words are different. Such differences can be attributed to the different motions of the throat for different words. Note that different words have different waveforms in pronunciation, which can introduce different motions of the throat with skin tugging and deformation states for the piezoresistive sensor.

A piezoresistive sensor was attached to a loudspeaker that played different words and phrases (“flexible”, “sensor”, and “flexible sensor”). Figure 7c shows the changes in the ratio of Δ*R*/*R*_0_ for the responses of the piezoresistive sensor to the sounds. There are distinct peaks in the ratio of Δ*R*/*R*_0_ in response to the corresponding syllables of the word. The three peaks of the relative change in the ratio of Δ*R*/*R*_0_ for the word “flexible” correspond to its three syllables of “fle”, “xi”, and “ble”; the two peaks of the relative change in the ratio of Δ*R*/*R*_0_ for the word “sensor” correspond to its two syllables “sen”, and “sor”. There are only four peaks in the relative change in the ratio of Δ*R*/*R*_0_ for the phrase “flexible sensor”, which can be attributed to the word linking.

**Figure 8:**
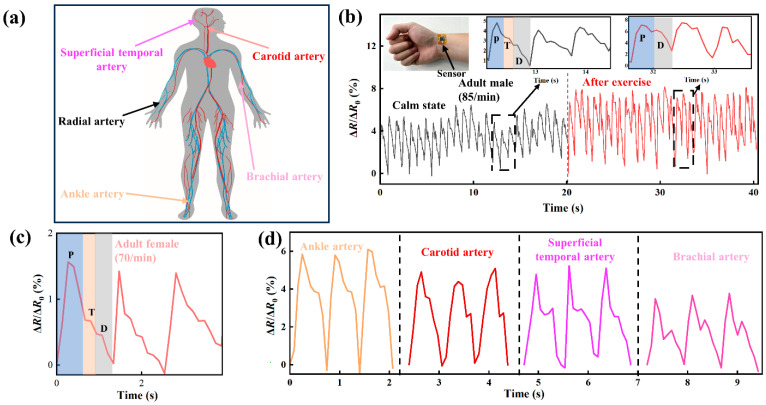
(**a**) Schematic of various arteries in the human body; temporal variations in the ratio of Δ*R*/*R*_0_ for the response of a piezoresistive sensor to the pulses of various arteries in different volunteers: (**b**) wrist pulses of a male adult in a calm state and 3 min after exercise(P,T,D are the shock, tidal and diastolic waves of the pulse, respectively) (**c**) wrist pulses of a female adult in a calm state, and (**d**) pulses of superficial temporal brachial, ankle, and carotid of a male adult.

A piezoresistive sensor was used to detect the epidermal pulse of a volunteer that is generated by the periodic systole and diastole of the heart. The epidermal pulse can be used to analyze the physical and pathological states of a person for clinical diagnosis in traditional Chinese medical science. The piezoresistive sensor can be attached to any place near the arteries of a person, as shown in Figure 8a, with medical PU tape.

Figure 8b presents temporal variations in the ratio of Δ*R*/*R*_0_ for the responses of a piezoresistive sensor, which was attached to the arm of a male adult near a radial artery in the calm state and 3 min after aerobic exercise. Multiple peaks are presented, and each peak consists of both ascending and descending branches due to the pulses of the radial artery. The piezoresistive sensor has a sufficient frequency–response bandwidth to capture the pulse signal, since the three characteristic waves, namely the shock wave (P-wave), tidal wave (T-wave), and diastolic wave (D-wave), of the descending branch of the beating pulse can be clearly identified. Each peak (cycle) takes about 0.7 s (i.e., 85 pulses per minute) for the calm state. For the pulses for 3 min after aerobic exercise, each peak can be divided into two regions corresponding to a P-wave and D-wave without a T-wave. Each peak (cycle) takes a less time, ~0.62 s (i.e., 97 cycles per minute). The clear difference in the characteristic waves between the calm state and the state after exercise indicates the great capability (or sensitivity) of the piezoresistive sensor to detect any subtle changes in the pulse. Both the frequency and strength of the ratio of Δ*R*/*R*_0_ increase after exercise compared to the corresponding frequency and strength of the ratio for the calm state. Such behavior is attributed to muscle artery dilation and vasodilation induced by exercise. To examine the reusability of the prepared piezoresistive sensor, the piezoresistive sensor was attached to the arm of a female adult near the radial artery for the calm state. Figure 8c shows that the piezoresistive sensor can successfully measure the pulses of the adult female. The three characteristic waves can clearly be identified, and it takes about 0.86 s per cycle for the calm state (i.e., 70 pulses per minute), demonstrating the reusability of the prepared piezoresistive sensor.

The responses of the piezoresistive sensor to the pulses of superficial temporal artery, carotid artery, brachial artery, and ankle artery were evaluated. Figure 8d shows temporal variations in the ratio of Δ*R*/*R*_0_ for the responses of a piezoresistive sensor that was attached to the skin of a volunteer in a calm state near a superficial temporal artery, carotid artery, brachial artery, and ankle artery. It is evident that each artery has its distinctive characteristics for the ratio of Δ*R*/*R*_0_, even though they have almost the same frequency. The peak at the ankle artery is higher than at the brachial artery due to the body weight supported by the ankles.

**Figure 9:**
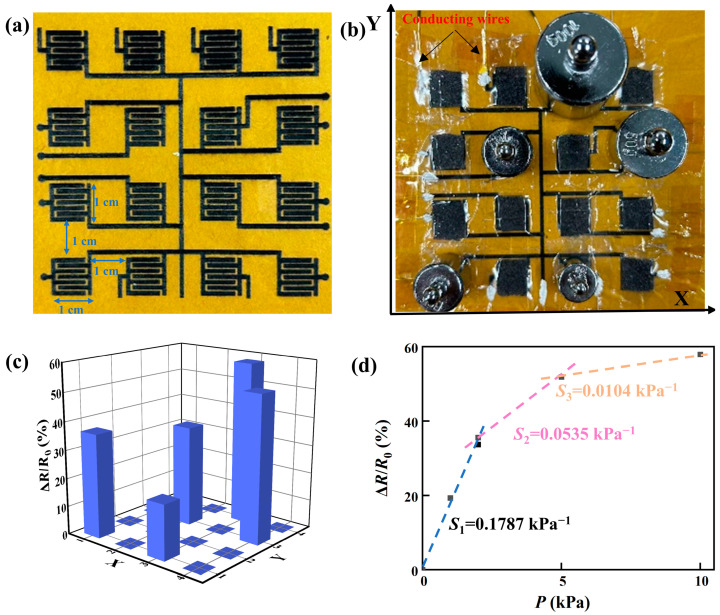
(**a**) A 4 × 4 array of piezoresistive sensors with 10 × 10 mm^2^ in size for individual sensors; (**b**) four different weights placed at five different nodes (two nodes have the same weight) of the 4 × 4 array of the piezoresistive sensors; (**c**) spatial distribution of the ratio of Δ*R*/*R*_0_; and (**d**) variations in the ratio of Δ*R*/*R*_0_ with applied pressure from the data in (**c**).

Following the approach of fabricating the piezoresistive sensors, we constructed a 4 × 4 array of piezoresistive sensors with 10 × 10 mm^2^ in size for individual sensors, as shown in Figure 9a. Four different weights of 10, 20, 50, and 100 g were placed at five different nodes of the array of the piezoresistive sensors, as shown in Figure 9b, and two nodes had the same weight of 20 g. Figure 9c shows the spatial distribution of the ratio of Δ*R*/*R*_0_, corresponding to the spatial distribution of the weights. There is only a slight difference in the ratio of Δ*R*/*R*_0_ for the same weight, suggesting that the array of the piezoresistive sensors can be used to measure the spatial distribution of pressure or mechanical loading, if the sizes of individual piezoresistive sensors can be further reduced. From Figure 9c, we obtain the sensitivities of individual piezoresistive sensors of the 4 × 4 array of the piezoresistive sensors, as shown in Figure 9d, to be 0.1787 kPa^−1^ at low pressures (0–2 kPa), 0.0535 kPa^−1^ at intermediate pressures (2–5 kPa), and 0.0104 kPa^−1^ at high pressures (5–20 kPa), which are in accord with the results shown in Figure 4a,b.

## 5. Summary

In summary, we successfully constructed flexible piezoresistive sensors from LIG electrodes and graphene-coated PDMS films with ridge-like surface structures. A simple and facile method was developed to produce the ridge-like surface structures from sandpapers via a replication strategy. The piezoresistive sensors exhibit Ohmic characteristics under mechanical loading, and the resistances of the piezoresistive sensors decrease with an increase in mechanical pressure, attributed to increases in the number of conducting pathways and the contact area under mechanical loading. At high pressures, the possible conducting pathways are exhausted, and the resistance decreases approximately linearly with increasing pressure.

The piezoresistive sensors made from the PDMS film, whose ridge-like surface structure was prepared with sandpaper of 93 μm in average particle size, exhibited the highest sensitivity and long-term stability, and were used in a variety of applications, including finger press, mouse clicking, and outgassing of an air-filled balloon. We demonstrated the feasibility of the piezoresistive sensor in the sensing of bio-related activities, such as muscle contraction, finger flexion, wrist flexion, elbow bending, knee bending, swallowing, respiration, sounds, and pulses. A 4 × 4 array of the piezoresistive sensors was constructed that was able to detect spatial distributions of external loading.

The approach developed in this research has opened a frontier to simply constructing flexible piezoresistive sensors for biomedical applications and wearable electronics. The research also reveals that one can tune the surface structures of flexible substrates in controlling the performance and sensitivity of the associated devices and systems.

## Figures and Tables

**Table 1:** Performance of pressure sensors prepared with sandpapers as replicated structures and with different sensing materials.

Sensing Material	Sensitivity (kPa^−1^)	Pressure Range (kPa)	Response/Recovery Times (ms)	Limit of Detection	Reference
Graphene	0.1667	0–2	47/8	96 mg	This study
	0.035	2–5			
	0.006	5–10			
AgNWs	0.1194	0–1.3	50/NA	NA	[56]
	0.0523	1.3–4			
Nanotubes	0.087	0–6.1	40/117	50 Pa	[57]
	0.009	6.1–29.1			
MWCNTs/CNT	0.024	1–20	72/105	NA	[59]
	0.0058	20–60			
CNT	−0.209	<1	195/158	5.0 Pa	[60]
CNTs	78.04	0–3	25/590	NA	[61]
	145.706	3–10			
	10.79	10–500			
MXene	281.5	0.36–2.34	67.3/44.8	7.8 Pa	[62]
	509.8	2.34–4.57			
	66.7	4.57–19.73			

## Data Availability

The data is available upon request via email.
